# Why Did Our Trial Not Work Out? A Qualitative Analysis

**DOI:** 10.32872/cpe.12887

**Published:** 2024-06-28

**Authors:** Eva Heim, Bleta Ademi, Ardiana Dacaj, Nadine Hosny, Sebastian Burchert, Arlinda Cerga Pashoja, Anna Hoxha, Mirëlinda Shala

**Affiliations:** 1 Institute of Psychology, University of Lausanne, Lausanne, Switzerland; 2 Department of Education and Psychology, Division of Clinical Psychological Intervention, Freie Universität Berlin, Berlin, Germany; 3 Faculty of Population Health, London School of Hygiene and Tropical Medicine, London, United Kingdom; 4 St Marys University, Twickenham, London, United Kingdom; 5 Department of Psychology, University of Zurich, Zurich, Switzerland; 6 Department of Economics, Lucerne University of Applied Sciences and Arts, Lucerne, Switzerland; Friedrich-Alexander-Universität Erlangen-Nürnberg, Erlangen, Germany

**Keywords:** cultural adaptation, online self-help, Albanians, ethnic minorities, access to care, recruitment

## Abstract

**Background:**

An online self-help programme for the treatment of depression called Hap-pas-Hapi was tested among Albanian-speaking immigrants in Switzerland and Germany, and two different levels of cultural adaptation were compared. Despite a massive recruitment effort, an insufficient number of participants could be recruited, and the drop-out rate was over 90%.

**Aims:**

We conducted a qualitative study to better understand the reasons for the non-use of Hap-pas-Hapi.

**Method:**

Eleven interviews were conducted with 17 Albanian-speaking participants aged 19-59. Participants were recruited for the purpose of this study and were not participants from the trial. They went through the recruitment material and the Hap-pas-Hapi introduction module, commented on the graphic design, usability, content, and shared their views about mental health and self-help.

**Results:**

Participants criticised the lack of a “design system” (i.e., a clearly identifiable and consistent graphic design) on social media for Hap-pas-Hapi, and the recruitment messages were unclear. The programme itself was perceived to be important and helpful for the community at large, but most participants said that they would not use it for themselves. The younger generation would have preferred an application in German or French, while the older generation did not see a benefit in using an online self-help programme to manage their psychological distress. Negative beliefs about mental disorders and psychological interventions were perceived to be common in this target group.

**Discussion:**

A professional recruitment strategy, a more careful selection of the target population (e.g., age groups) and different kinds of adaptations might have resulted in a better acceptance of the intervention. At the same time, anti-stigma campaigns and psychoeducation are needed to enhance treatment motivation.

## Highlights

- Our randomised controlled trial among Albanian-speaking immigrants encountered major challenges.
- Considering different age groups within the target population might have made a difference.
- A professional recruitment strategy and a consistent graphic design on social media was suggested.
- Negative beliefs about mental health services need to be addressed in the target population.

## Background

We recently published the results of a randomised controlled trial (RCT) that was conducted among the Albanian-speaking population in Switzerland and Germany (Heim et al., 2024, this issue). In this RCT, we aimed to compare two levels of cultural adaptation of an online self-help intervention called Hap-pas-Hapi (Albanian for Step-by-Step) for the treatment of depression (Carswell et al., 2018; Shala et al., 2020a). A massive recruitment effort through different channels resulted to be ineffective: Instead of the targeted *N* = 320 participants, we were able to recruit *N* = 97 (completed baseline assessments). More than half (56%) of the consented participants did not complete the baseline assessments. Furthermore, drop-out rates (completed post-assessment) were over 90% in both treatment conditions (i.e., surface vs. deep structure level adaptation), with no observed differences between groups.

There are several potential explanations for these difficulties. First, it is possible that the recruitment strategy did not reach the community. Second, it could be that the recruitment strategy reached the community, but it was not clear what Hap-pas-Hapi was, or how it could be beneficial. And third, we must also consider the possibility that the recruitment strategy was seen and was properly understood by the community, but Hap-pas-Hapi may not fulfil the needs of this particular target population.

There are compelling reasons to reject the first hypothesis. We achieved up to 23,000 reaches through our Facebook ads. The events (online and face-to-face) that were organised among Albanian associations (see Heim et al., 2024, this issue) were well attended, and we received very positive feedback about the project and Hap-pas-Hapi. Albanian-speaking health professionals in Switzerland and Germany promoted the intervention among their patients and within their networks, and a group of “cultural brokers” (Wenger, 1998) tried to reach participants outside the health sector. Thus, given the resources and efforts put into promoting the study, it seems unlikely that the Albanian community would have missed information on the project or the opportunity to sign up.

As a second option, Hap-pas-Hapi, the study, or some related aspects might have been misconceived or misinterpreted by the community. In other words, our recruitment strategy and the introduction module of the intervention might have missed the central messages, might have contained messages that were not relevant, or might even have caused resistance or mistrust. For this option, it is important to consider that the Albanian community in Switzerland and Germany is highly diverse, including people from different countries of origin who immigrated for a variety of reasons, including labour migration, family reunion and armed conflicts (Shala et al., 2020b). Second-generation immigrants differ from first-generation immigrants regarding their relationship with Albanian culture, their integration in Switzerland, their expression of psychological distress, and their attitudes towards mental health services (Pnishi et al., 2024). All these aspects must be considered when reflecting about messages and contents of recruitment materials and the platform.

The third explanation, i.e., that Hap-pas-Hapi is not relevant for the community or does not correspond to a real need, must be considered, as well. There are some indications suggesting that this is not the case. In a representative study, the Swiss Health Observatory (obsan) found that the risk for mental distress was twice as high (relative risk = 2.0) among first-generation immigrants aged 50-64 than among natives of the same age group. Our own ethnopsychological studies also showed pronounced psychological distress among Albanian-speaking individuals in Switzerland (Pnishi et al., 2024; Shala et al., 2020b). In addition, the online self-help intervention has shown to be acceptable and efficacious in two large-scale RCTs in Lebanon among Syrian displaced people and the Lebanese population (Cuijpers et al., 2022a; Cuijpers et al., 2022b). In these trials, more than 1200 participants were recruited within six months, during the Covid pandemic and amidst major economic, social, and political turmoil. Drop-out rates (completed post-assessment) were much lower, i.e., 65% in the Lebanese population (Cuijpers et al., 2022a) and 46% among Syrian refugees (Cuijpers et al., 2022b). In summary, there seems to be a need in terms of psychological distress in the target population, and the intervention itself was tested successfully under challenging conditions in a different setting.

Hence, to better understand the reasons and factors that contributed to the low uptake and high attrition in our trial, we conducted a qualitative study with Albanian-speaking participants in Switzerland who participated in semi-structured interviews. In our ethnopsychological studies that were conducted prior to adapting Hap-pas-Hapi, we had identified clear differences between the older first-generation immigrants, and the younger second-generation group, regarding their cultural concepts of distress and treatment expectations (Pnishi et al., 2024; Shala et al., 2020b). Therefore, we aimed to include both groups in this post-hoc qualitative study, to better understand their views.

## Method

### Participants and Procedures

Seventeen (eight female and nine male) participants were interviewed in this study. Inclusion criteria were: Albanian origin (i.e., Albania, Macedonia, and Kosovo); understanding of Albanian language; age 18-65. The participants’ age range was 19-59 years old where nine individuals were 19-27 years old, and eight were between 45-59 years old. Participants were recruited by two Albanian-speaking Master students at the University of Lausanne through their respective social networks including Facebook, Instagram, e-mails, and face to face communications. Some of the participants were personal acquaintances of the two Master students who conducted the interviews. Most interviews were conducted in French and two in Albanian language. Participants were informed about their voluntary participation, their right to withdraw from the study without giving any reasons, data protection, and the use of the results. They signed an informed consent form before starting the interview. Participants received a voucher of CHF 50 for their time and travel due to participation in the study. The study was revised and approved by the ethical review commission of the University of Lausanne (E-SSP-072O22-OO).

BA and AD conducted 11 interviews: four were conducted with pairs, i.e., participants of two different age groups; one with three participants and six were individual interviews. We anticipated that discussions among two participants of different age groups could reveal potentially diverging views. By contrast, the method of larger focus groups was not deemed efficient for logistical reasons, as participants had to go through a lot of material (recruitment and parts of the platform), which would be difficult to realise in a timely manner with larger groups.

### Interview Guide

The interview guide contained three parts: First, participants commented on the recruitment material, i.e., Facebook and Instagram ads, flyers, and a promotional video. All materials except the flyers were shown on a Tablet, and participants were free to browse through the materials for as long as they needed. The flyers were printed so that participants reviewed them in paper format. Participants were asked to express their opinion about the materials (e.g., “what do you think about these posts?”, see Heim et al., 2024S).

Second, they reviewed the introduction session of the Hap-pas-Hapi programme online, and were invited to provide feedback on its content, graphic design, and usability. We decided to focus on the introduction session, since in the RCT (Heim et al., 2024), less than 50% had completed it. Although dropout rates had been equal between the two levels of cultural adaptation in the RCT, we showed participants the deep structure adaptation in this post-hoc, qualitative study. This adaptation is described elsewhere (Shala et al., 2020a). The adapted introduction session contains a narrative and an exercise part. In the narrative part, a main character (male or female) gives an illustrated account of their history of depression, how they sought help and started therapy with a doctor. The doctor (who wears a white coat, see Abi Ramia et al., 2018) provides psychoeducation and recommends psychological exercises. All parts are presented online either as text or as audio recordings. In the exercise part, participants are asked to complete lists with their own symptoms and perceived causes for distress. They can choose Albanian idioms of distress (e.g. *mërzi, vuajtie*) and potential causes (e.g., family problems, fatalistic beliefs about symptoms) from drop-down lists or write down their own idioms of distress (Shala et al., 2020a). Participants in our study went through this introduction section, and their behaviour was observed while navigating through the platform and were invited to “think aloud” (Willis, 2004). Interview questions addressed the content of the narrative part and the exercises (see Heim et al., 2024S)

In the third part of the interview, participants were asked about their cultural beliefs related to mental health in general. Interviews lasted between 50 and 120 minutes each. An interview question was, e.g., “how would you describe mental health issues and attitudes towards mental health services in the Albanian-speaking community?” (see Heim et al., 2024S).

### Data Analysis

We conducted thematic analysis (Braun & Clarke, 2006), which includes six phases. BA and AD transcribed the data and read all the transcripts several times to familiarise themselves with the data. They created a first set of inductive codes using MAXQDA 2020 (VERBI Software, 2017). Codes were then grouped into themes by the same authors. This initial coding frame was revised in the larger research group (BA, AD, EH, and NH), before all interviews were coded by BA and AD.

## Results

Results are presented along the interview guide structure. First, we present results related to recruitment materials, followed by results concerning the Hap-pas-Hapi introductory session. Lastly, we summarise participants’ cultural beliefs concerning mental health.

### Recruitment Materials

After looking through the social media ads, participants agreed that Hap-pas-Hapi’s “design system” on social media was inconsistent, and not sufficiently convincing. One participant perceived the communication strategy as being “under construction”, and another as “work in progress”. They suggested using a design system with consistent colours, as it has been done for the platform itself. As to the other materials, the flyer was perceived to be overloaded with information.

The content of the ads was also criticised. Participants said that the information provided through different ads was too diverse, and that important information was missing (e.g., content and aims of the app). For example, a simulated exchange on WhatsApp, in which one person recommends Hap-pas-Hapi to another, apparently gave the impression that Hap-pas-Hapi was based on an actual exchange between people, not a self-help platform. Others mistook it for a platform for exchanging messages with a psychologist.

Participants also expressed their doubts about the potential benefits of using Hap-pas-Hapi (“I really don’t understand how it will help me”). They asked questions about the target population (“Who is this for?”), and the younger participants expressed doubts that the generation of their parents (i.e., first-generation) would apprehend the Facebook ads or download a self-help app on their mobile phones. They would rather use WhatsApp and Viber to communicate with their family who are often dispersed across different countries. This lack of “digital literacy” was mentioned frequently as one major barrier to a wider distribution and use of Hap-pas-Hapi.

Mistrust was another barrier observed recurrently by participants. Some of them expressed that they would never download an app just because they had seen a Facebook ad, and they asked questions about data use and protection. They suggested that ads should focus much more on privacy and data protection, to make sure people would trust before they downloaded the app. One participant said that the idea of receiving 30 CHF for participating in the study provoked a feeling of being instrumentalized for research purposes, especially if the social media campaign was perceived to be “work in progress”. This gave him the impression that he was used for something that was not fully developed.

Participants of both generations also wanted to know more about the creators of Hap-pas-Hapi, and the concept behind it. They thought that people would need more information about its use and benefits before embarking on it. A picture showing researchers “behind the scenes” was unanimously perceived to be a good strategy for reaching people’s attention, as it illustrated the (Albanian speaking) researchers’ academic career, and therefore increased trust in the study. Participants also suggested posting statements by real people who have used Hap-pas-Hapi and recommend it, or using ambassadors, influencers, celebrities, who can promote the platform more efficiently and increase trust.

### The Hap-pas-Hapi Programme

Hap-pas-Hapi was perceived to be relevant, well developed, and professional, which stands in contrast to the perception of the recruitment strategy. However, most participants felt that the application’s interface was not clear enough. In nine out of eleven interviews, participants mentioned difficulties in understanding the instructions and navigating through the platform. These difficulties were even more pronounced in the older age group. By consequence, many participants expressed annoyance, impatience, or frustration when they did not understand how to continue, or when a lot of apparently irrelevant information was presented. Even the personal stories provided in the intervention were perceived to be cumbersome by some of them. Participants suggested to jump right to the exercises, and not to make people read or listen to the story of the main character. They also preferred the easier exercises (e.g., a grounding exercise) to the more complex ones (e.g., planning an activity with several steps).

One important result concerned the narratives of the main characters in the application. In the older age group, several participants expressed difficulties in relating to this story. They felt that the narratives caused a certain feeling of “being exchangeable” and a lack of taking their own history and emotions seriously. One participant said:

“…to say that in fact a quarter of the world’s population is affected by this, that can be good because it puts the problem into perspective, we tell ourselves that we are not the only ones. But at the same time, it also takes away the personal side, of saying, well, once again, I am one of the two billion, and they are not going to look after me, because they are not going to look after the two billion.”

The graphic design was positively commented. Some participants liked the illustrations, others disliked them, which is to be expected as it corresponds to personal taste. Participants’ opinions regarding the doctors’ white coat were also diverse. Three participants from the younger generation thought that the white coat was unnecessary, whereas one participant from the older generation considered it to be important. The others did not comment on the doctors’ white coat. As to the audios, they were appreciated by the younger generation, but the older generation preferred reading. Language was another frequently mentioned topic. First, the younger generation would have preferred the option to choose between Albanian and a Swiss language (i.e., French or German). Second, since it had not been possible to accommodate the two Albanian dialects (Gheg and Tosk), the standard Albanian language was used in Hap-pas-Hapi (see Shala et al., 2020a), which caused controversial discussions. While some of them thought that the language was well chosen, others disagreed. This is illustrated in the citation below:

“…it can be a problem for some people, but it's always a problem because you can't adapt to both populations, those who speak well and those who don't understand. Intrinsically, from the outset the project starts with a limit.”

### Cultural Beliefs Related to Mental Health

The last part of the interview guide revealed important insights about perceptions and attitudes related to mental health. Participants mentioned a high stigmatisation of mental disorders in the community, and a lack of mental health literacy, which prevented people from seeking help. Many said that Hap-pas-Hapi was a great platform and much needed by the community (they would even recommend it to other people), but at the same time, they all said that they would not use it personally, as they were not faced with mental health problems. One participant also said that people would not use the platform because they would not want to be associated with “crazy” people.

Explicitly expressing distress was perceived as being difficult, as people would often not have words for their feelings. By consequence, participants perceived a lack of introspection in their own community. One participant said that this caused difficulties in using Hap-pas-Hapi, as people would not know which option(s) to choose (e.g., regarding symptoms), and some might even have difficulties in understanding the word “symptom” in the first place.

## Discussion

This qualitative study revealed important insights on reasons for our difficulties with recruitment and adherence in the Hap-pas-Hapi randomised controlled trial in Switzerland and Germany (Heim et al., 2024, this issue). First, participants strongly criticised the recruitment material. The flyer was perceived to be overloaded with written information, which can be explained by restrictions concerning recruitment materials as imposed by ethical standards. Participants also criticised the messages and pictures promoted on social media such as Facebook and Instagram, and particularly the lack of a clear “design system” in the sense of a consistent graphic design. This is in contrast with the recruitment strategy that was used in Lebanon, where a professional agency was hired to implement the social media campaign (Heim et al., 2021). A difficulty also comes from the fact that, for ethical reasons, we promoted participation in a study, and not the app itself.

In summary, these results suggest that it is worth collaborating with a professional promotional agency, and investing financially, not just towards the online platform, but also (quite substantially) towards recruitment. It is important to create clear and consistent messages to inform the audience about the app, potential benefits of participating in the study and of using the app, as well as about data use and protection. In addition, our results suggest that personalised recruitment works best, as the picture showing researchers “behind the scenes” was well received. In Lebanon, personalised recruitment, e.g., through WhatsApp broadcasts sent by United Nations High Commissioner for Refugees (UNHCR), seemed to be efficient (Heim et al., 2021). We can learn from these experiences and use it for future trials.

On the other hand, would it really have made a difference if we had invested in a professional recruitment strategy? Maybe we would have reached a wider population. However, there is strong indication that this would not have been sufficient. We have anecdotal and empirical evidence from the present study that Albanian-speaking individuals in Switzerland perceived the app to be very relevant and useful – for others; that is, for those who suffer from psychological distress, but not for themselves. This strong division into “them” and “me” reflects the stigma related to mental disorders in the Albanian community (Thornicroft et al., 2022). Is this stigmatisation stronger than in other communities? We have no evidence, but our results suggest that there is a strong resistance against using mental health care, even if it comes in the form of an anonymous self-help app that can be used in private.

It is possible that anti-stigma campaign before launching Hap-pas-Hapi, or in parallel, could have been effective. Strong beliefs in suffering being part of one’s life and destiny which has to be endured with patience, may prevent people from seeking professional help (Shala et al., 2020b). We aimed to address these beliefs with a deep structural adaptation of the Hap-pas-Hapi introduction session, by using culturally relevant terms, expressions, sayings, and beliefs, and using exercises to challenge these beliefs (Shala et al., 2020a). We used an adapted version of an intervention that has shown to reduce fatalistic beliefs and enhance treatment motivation among Turkish participants in Germany (Reich et al., 2021). It seems that in our sample, this intervention was not robust enough, as it did not result in less drop-outs than in the group who received the standard Hap-pas-Hapi without the culturally adapted introduction (Heim et al., 2024, this issue). Cultural concepts related to emotions and emotion expression in the community – i.e., the fact that one’s own emotions and inner life are not supposed to be expressed explicitly – might be one major barrier to using the application.

As to the feedback on the platform itself, most of it corresponds to what has been found in other qualitative studies about Step-by-Step (Abi Ramia et al., 2018). Despite this feedback, the app was used more frequently among different populations in Lebanon, while the Albanian version remained unused. We have some indication that we missed the target population because we did not pay sufficient attention to the different generations within the Albanian community. The younger generation who in general is more open towards mental health treatments and online self-help applications consistently said that they would have preferred an application in German or French, or the option to choose the language. In the Albanian version, the use of a specific dialect of one sub-group may cause resistance in another sub-group within the target population.

As a further difficulty, the older generation, for whom the intervention was mainly adapted (e.g., by including Albanian concepts of distress) was not sufficiently “tech-savvy” to use an application for their mental health. In our planning, we thought that an app would help overcome stigma and the barriers of seeking help. But it seems that with a technology-based intervention, we even added another barrier, because the digital skills and interest is limited in the group of first-generation immigrants to whom we adapted the content. Promoting digital health literacy in this population could increase motivation for treatment and thus improve the accessibility and effectiveness of such interventions in the future.

Even though it is suggested that clinical research in high-income countries is not sufficiently “inclusive” when it comes to ethnic minorities (Hussain-Gambles et al., 2004; Wendler et al., 2006), it rather seems that in our target population, there was some kind of “auto-exclusion” from research, due to mistrust and the feeling of being instrumentalised, which leads to a lack of interest or motivation to contribute to research. We observed a strong mistrust in the platform itself, data use and protection, the purpose of this research, and the benefit for oneself in using Hap-pas-Hapi. From our research in this community (Pnishi et al., 2024; Shala et al., 2020b), it seems that people are enough burdened with their own daily lives and struggles, and Hap-pas-Hapi was not perceived as something that could help with this, but rather as a tool without considerable potential benefit. The fact that 56% of participants dropped-out during baseline assessments (Heim et al., 2024), and the negative comments about the intervention in the present study, strongly suggest that this was the case.

What lessons can be learnt from this difficult endeavour? It seems that despite a massive effort of conducting an ethnopsychological study among the target group, a meticulous cultural adaptation, and a carefully developed recruitment strategy, engaging our hard-to-reach target group has proven to be more difficult than expected. The language skills and cultural knowledge of our extensive research team, composed primarily of Albanian-speaking members, were surely and asset, but this was still insufficient to motivate the larger community to engage in our project.

Some limitations to this study are related to sampling, as participants were recruited in the social networks of two authors (BA, AD), and some were their acquaintances. This might have influenced the responses during the interviews. However, this is not necessarily a limitation, since the pre-existing rapport between the authors and some participants could have led to more uninhibited and in-depth accounts and might have given researchers the ability to delve deeper into topics and prompt participants more effectively, thereby eliciting richer and more relevant insights. Furthermore, participants in the present qualitative study had not participated in the randomized controlled trial themselves. It would have been interesting to interview those who dropped out, but it was very difficult to reach them once we lost contact with them. On the other hand, having a “fresh view” on the recruitment materials and the introductory module may have delivered more valid data. We also did not assess participants’ level of depression, which might have been relevant for interpreting our data. The two interviewers, who also conducted the analyses, both speak Albanian and French, which can be viewed as a strength of our study.

As we reflect on our approach, we identify potential avenues for optimising future efforts. Most likely, we should have targeted the younger generation with an application in the local language (German or French) and address their needs more specifically, rather than carefully adapting the programme to the cultural concepts of distress of first-generation immigrants who would not use such a programme in the first place. For those, a different kind of approach would be needed, most likely based on direct human contact. Furthermore, an anti-stigma campaign before promoting an app could help address the major barriers in this community. Such campaigns, if well planned and implemented, are effective, as evidence shows (Thornicroft et al., 2022). We hope that in the future, other researchers can benefit from our experience, and more inclusive interventions can be developed for minority groups who are in need of mental health care.

## Supplementary Materials

The Supplementary Materials contain the following items (for access, see Heim et al., 2024S):

- **Interview guide:** Questions related to the recruitment material and the Hap-pas-Hapi self-help programme.
- **Coding framework:** Themes and sub-themes used in the data analysis process.

HeimE.
AdemiB.
DacajA.
HosnyN.
BurchertS.
Cerga PashojaA.
HoxhaA.
ShalaM.
 (2024S). Supplementary materials to "Why did our trial not work out? A qualitative analysis"
[Interview guide and coding framework]. PsychOpen. 10.23668/psycharchives.14648
PMC1130391639119055

## Data Availability

The dataset is available upon request.

## Reporting Guidelines

The study followed the Consolidated criteria for reporting qualitative research (COREQ, Tong et al., 2007).
